# PREVALENCE AND PAYMENTS PER CAPITA OF ADRD IN PUERTO RICO AND THE US: ESTIMATES FROM MEDICARE DATA

**DOI:** 10.1093/geroni/igad104.2756

**Published:** 2023-12-21

**Authors:** Jose Carrion-Baralt

**Affiliations:** University of Puerto Rico Medical Sciences Campus, San Juan, Puerto Rico, United States

## Abstract

Puerto Rico is one of the oldest US jurisdictions (22.7% aged 65 and over – 65+), where over 40% of the 65+ population lives under federal poverty levels. However, very little epidemiological and health economics research on Alzheimer’s Disease and Related Dementias (ADRD) has been conducted on this population. This work compares prevalence of ADRD and several of its risk factors, as well as Medicare Payments-Per-Capita (PpC) between the Puerto Rico (PR) and US National populations aged 65+, and between males and females 65+ in PR. We compared prevalence and PpC data for PR and US for ADRD and Diabetes, Hypertension, Hyperlipidemia and Depression from the publicly available CMS Multiple Chronic Conditions Data Warehouse 2018 file. All fee-for-service beneficiaries were included. Prevalence of ADRD in PR was 33% higher than in the US, while Medicare PpC were 146% higher in the US. Prevalence of Diabetes in PR was 77% higher than in the US, while Medicare PpC were 80% higher in the US. Prevalences of hypertension and hyperlipidemia were not significantly different, but PpC were almost double in the US. Prevalence of depression was 30% lower in PR. Prevalences of ADRD and all other conditions were higher among PR women than among PR men. Significant health disparities exist between PR and the US (and between PR males and females), both in terms of prevalence and PpC of ADRD and several associated risk factors. These findings are important evidence for the formulation of public policies that can help eliminate these disparities.

